# Acceptance of universal varicella vaccination among Swiss pediatricians and general practitioners who treat pediatric patients

**DOI:** 10.1186/s12879-020-05586-3

**Published:** 2021-01-06

**Authors:** Florian Lienert, Olivia Weiss, Kathrin Schmitt, Ulrich Heininger, Patrik Guggisberg

**Affiliations:** 1 MSD Merck Sharp & Dohme AG, Lucerne, Switzerland; 2 Current affiliation: Bavarian Nordic AG, Zug, Switzerland; 3 IPSOS, Basel, Switzerland; 4 Department of Pediatric Infectious Diseases and Vaccinology, University of Basel Children’s Hospital, Basel, Switzerland

**Keywords:** Varicella zoster virus, Varicella, Chickenpox, Vaccination, Survey, GP, Pediatricians, Switzerland

## Abstract

**Background:**

Over the last two decades, several countries have initiated universal varicella vaccination (UVV) programs in infants. In 2019, the Swiss National Immunization Technical Advisory Group (NITAG) decided to start evaluating the introduction of universal varicella vaccination. There is a theoretical concern that suboptimal vaccination coverage could lead to a shift in the varicella incidence to older age groups, thereby potentially increasing complication rates. To achieve a high vaccination coverage rate, it is important that practicing physicians comply with a potential recommendation for UVV.

We studied the perception of varicella and the current vaccination behavior among Swiss pediatricians and general practitioners (GPs) who treat children. We also assessed their intention to advise parents to vaccinate their children against varicella in the event the Swiss NITAG will recommend UVV.

**Methods:**

Primary data was collected through a structured, 20-min online survey with Swiss pediatricians and GPs who treat children.

**Results:**

150 physicians participated in the study: 40 GPs in the German-speaking part, 20 GPs in the French-speaking part, 67 pediatricians in the German-speaking part, and 23 pediatricians in the French-speaking part.

The majority (64%) of all participants reported that they currently recommend varicella vaccination for risk groups according to the national immunization plan. About one third of physicians (35%) – predominantly pediatricians – currently already recommend it for all infants. In these situations, a measles, mumps, rubella, varicella combination vaccine is currently used by 58% for the first dose and by 59% for the second dose.

86% of participants stated that they would advise parents to have their children vaccinated against varicella in case of a recommendation for UVV by the Swiss NITAG. 68% responded that they expect many questions from parents and 65% agreed that they have good arguments to convey the importance of varicella vaccination.

**Conclusions:**

The survey study results show that most participating pediatricians and GPs indicated a favorable attitude towards childhood vaccination against varicella in the setting of a Swiss NITAG recommendation for UVV. This data shows the importance of NITAG recommendations in influencing vaccine education and supporting achievement of high coverage of varicella vaccination.

**Supplementary Information:**

The online version contains supplementary material available at 10.1186/s12879-020-05586-3.

## Background

Varicella is a common infectious disease that is caused by varicella zoster virus (VZV), which is transmitted from humans to humans by droplets [1, 2]. Varicella is typically a mild disease but can cause serious complications; the rate for complications in children and adolescents in Switzerland has been estimated at 1 hospitalization per 1000 varicella cases [3]. In adults the hospitalization rate is up to 10-fold higher [2].

In Switzerland, vaccinations are voluntary; the decision to vaccinate oneself and one’s children is each individual’s responsibility. An annual vaccination schedule serves as a national guideline and classifies recommended vaccinations as either “basic”, “supplementary”, or “risk-based” vaccinations. Vaccinations are defined as “basic” if considered essential for individual and public health. These must be recommended by physicians to their patients. Vaccinations that provide optimal individual protection and are intended for people who want to protect themselves against clearly defined risks, but are not considered essential for public health, are categorized as “supplementary”. Physicians in Switzerland must inform their patients of the existence of supplementary vaccinations. Universal vaccination against varicella during childhood is not recommended. The Swiss Federal Office of Public Health currently recommends varicella vaccination in individuals 11 to 40 years of age who have not contracted varicella naturally in the past (i.e. who do not have a reliable varicella history or are seronegative for VZV-IgG serum antibodies) as a “basic” vaccination. Immunization against varicella is also recommended as a “risk-based” vaccination for individuals from the age of 1 year onwards with an increased risk for complications or transmission [4]. As in most other European countries at a time before implementation of universal varicella vaccination, over 90% of the Swiss population has had varicella by the age of 15 years [5].

The United States of America was the first country to introduce a UVV program, starting with a 1-dose recommendation in 1995 and switching to a 2-dose recommendation in 2005. UVV has also been introduced in Germany as a 1-dose program in 2004 with a switch to a 2-dose program in 2009. In both countries, implementation of UVV resulted in a strong decline of the incidence of varicella and varicella-related hospitalizations and deaths [6, 7]. Italy started to progressively introduce UVV in different regions from 2003 on and switched to a national recommendation in 2017. Similarly, Spain progressed from UVV in a few regions to a national program in 2016 [8]. In both Italy and Spain, a progressive reduction in the incidence of cases and hospitalizations was found in regions that introduced varicella vaccination programs [9, 10]. While several countries have universal varicella vaccination programs in place, many postponed their introduction due to concerns about a shift in the disease to older groups, an increase in herpes zoster in the elderly, and cost-effectiveness [11]. The concern about an age shift is based on modelling studies that suggested a high vaccination coverage (> 80%) is needed to prevent an increasing incidence rate of complications due to the potentially resulting shift in the incidence to older ages groups [12, 13].

In Switzerland, there is no legal basis for mandatory vaccinations except for situations of major epidemics. Thus, mandatory vaccination is not a means to ensure a high level of varicella vaccination. To achieve a high varicella vaccination coverage rate, it is however important that practicing physicians comply with a potential recommendation for UVV. In this study, we therefore aimed at determining the perception of varicella and the current vaccination behavior among Swiss pediatricians and GPs who treat children. We also wanted to assess their intention to advise parents to vaccinate their children against varicella in the event the Swiss NITAG will recommend UVV.

## Methods

### Study population and design

Participants were recruited by QualiPro (https://qualipro.ch/index.html), an agency specialized in Swiss healthcare professional recruitment for surveys, from their existing database. Based on screening questions, only office-based physicians from the German- and the French-speaking parts of Switzerland were selected. To qualify for participation, physicians had to be practicing in their specialty between 2 and 35 years and currently vaccinating children.

Based on previous experiences with unpublished surveys among physicians in Switzerland, a sample size of 130–150 is achievable in terms of recruitment and has proven to provide robust results. Therefore, the following set of sample quotas was defined: 35–50 GPs in the German-speaking part, 15–20 GPs in the French-speaking part, 60–75 pediatricians in the German-speaking part, and 20–25 pediatricians in the French-speaking part. Once 150 participants responded, the recruitment was stopped.

In November 2019, 1′208 GPs and 1′054 pediatricians received an invite to participate in an online-based survey study. Reminders were sent one week later. The first 150 physicians who completed the screening questions and passed the screening criteria continued with the questionnaire. Once 150 eligible physicians completed the questionnaire, the link showed a quota full screen indicating that the survey has been closed and the link to the questionnaire was no longer active. Physicians who participated gave their informed consent by filling out the questionnaire.

The questionnaire (additional file 1) was similar to that used by other investigators on the same topic [14] and was adapted by the authors of this publication. It contained questions on background characteristics, knowledge about varicella, attitude towards universal varicella vaccination, and beliefs about the disease varicella and varicella vaccination. Most questions were phrased as statements on which the level of agreement was measured using a 5-point Likert scale.

### Data analysis

#### Attitude towards universal varicella vaccination

The primary outcome of the study was to understand physicians’ intention to advise parents to vaccinate their children against varicella in case the Swiss NITAG would recommend UVV. The respondents were asked to indicate on a 5-point Likert-scale whether they agreed with the following statement (1 = strongly disagree, 5 = strongly agree): “If the NITAG recommends varicella vaccination for all infants starting at the age of 9 (-12) months as a basic vaccination, I will advise parents to vaccinate their children against varicella”. The respondents were classified to have a positive attitude towards a recommendation if they selected 4 or 5 on the 5-point Likert-scale.

#### Determinants of attitude towards universal varicella vaccination

Knowledge about varicella:

We calculated a knowledge score that was based on six knowledge questions. The maximum of this score was 6 points (1 point per correct answer, see also additional file 1). Respondents were classified as having ‘limited knowledge’ (0–2 points), ‘moderate knowledge’ (3–4 points) or ‘good knowledge’ (5–6 points) about varicella. We tested differences in knowledge between physicians using Pearson’s χ2 or Fisher’s exact test. We applied the Benjamini-Hochberg method with a false discovery rate of 0.05 to correct for multiple testing [14, 15].

Beliefs about varicella and varicella vaccination:

To get insight into the perceived severity of varicella, participating physicians were asked to assess the importance of vaccination for children (from the approved age onwards) for different vaccine-preventable diseases including varicella. General beliefs and perceptions about varicella and varicella vaccination were measured by 7 statements: “Varicella can cause serious complications”, “Varicella generally has a mild disease course in healthy children”, “I believe that varicella is a burden to working parents and causes productivity loss”, “I think that varicella is a disease serious enough to vaccinate against”, “Varicella is a disease one should have experienced as a child (in order to develop specific immunity)”, “One should not keep children with varicella away from school or child care”, and “I am worried about potential side effects of the varicella vaccination”.

To study differences in beliefs between GPs and pediatricians mean scores and associated simultaneous Bonferroni confidence intervals with overall coverage of at least 95% were calculated.

Logistic regression analyses:

Logistic regression models were used to identify determinants for Swiss physicians’ intention to advise parents to vaccinate their children against varicella in case of recommendation for UVV through the Swiss NITAG.

The following potential determinants were included in the univariable and multivariable logistic regression analyses: gender, years of practice, specialty, varicella knowledge score and beliefs about varicella and varicella vaccination (see additional file 1 for more details on included questionnaire items). Only significant variables were kept in the model.

For these regression analyses, the agreement on statements regarding beliefs about varicella and varicella vaccination was divided into three categories: a) no agreement (‘strongly disagree’ or ‘disagree’), b) neutral (‘neutral’), and c) agreement (‘agree’ or ‘strongly agree’). A determinant was considered statistically significantly associated with the outcome if the *P* value was < 0.05. The logistic regression analyses were conducted using SPSS 24.0.

## Results

### Response and background characteristics

150 physicians participated in the study: 40 GPs in the German-speaking part, 20 GPs in the French-speaking part, 67 pediatricians in the German-speaking part, and 23 pediatricians in the French-speaking part. Their background characteristics are presented in Table 1. Overall, respondents included an equal number of male and female physicians. A large proportion had children of their own and all of them reported complete immunization of their own children according to the National Immunization Plan (NIP).

**Table 1:** Background characteristics of participating physicians

	Pediatricians (*N* = 90)		GPs (*N* = 60)		Total (*N* = 150)	
Background characteristics	N	% [95% CI]	N	% [95% CI]	N	%
**Region**						
German-speaking region of Switzerland	67	74.0% [65.4–83.5]	40	67.0% [54.7–78.6]	107	71.0%
French-speaking region of Switzerland	23	26.0% [16.5–34.6]	20	33.0% [21.4–45.3]	43	29.0%
**Sex**						
Male	34	37.8% [27.8–47.8]*	43	71.7% [60.3–83.1]*	77	51.0%
Female	56	62.2% [52.2–72.2]*	17	28.3% [16.9–39.7]*	73	49.0%
**Years in Practice**						
2–10 years	26	28.9% [19.5–38.3]	17	28.3% [16.9–39.7]	43	28.7%
11–20 years	47	52.2% [41.9–62.5]	26	43.3% [30.8–55.9]	73	48.7%
21–35 years	17	18.9% [10.8–26.9]	17	28% [16.9–39.7]	34	22.7%
**Have children on their own**						
Yes	75	83.0% [75.6–91.0]	52	87.0% [78.1–95.3]	127	84.7%
No	15	17.0% [8.9–24.4]	8	13.0% [4.7–21.9]	23	15.3%
**Have their children fully vaccinated as per NIP**						
Yes	75	100.0% [100%]	52	100.0% [100%]	127	100.0%
No	0	0%	0%	0%	0	0%

* significant difference between GPs and pediatricians (*p*-value < 0.05)

### Attitude towards universal varicella vaccination

Most physicians reported their intention to advise parents to vaccinate their children against varicella if universal varicella vaccination was recommended by the Swiss NITAG as a basic vaccination (Table 2).

**Table 2:** Perception and attitudes about a universal varicella vaccination

	Pediatricians (N = 90)		GPs (N = 60)		Total (N = 150)	
Questionnaire Item	N	%^a^ [95% CI]	N	%^a^ [95% CI]	N	%^a^
*If the NITAG recommends varicella vaccination for all infants starting at the age of 9 (−12) months as a basic vaccination, I will advise parents to vaccinate their children against varicella*	83	92.2% [86.7–97.8]*	46	76.7% [65.9–87.4]*	129	86.0%
*A strong recommendation from the NITAG to use the quadrivalent measles, mumps, rubella, varicella (MMRV) will lead to higher vaccination rates*	74	82.2% [74.3–90.1]	43	71.7% [60.3–83.1]	117	78.0%
*If the NITAG recommends varicella vaccination for all infants starting at the age of 9 (−12) months as a supplementary vaccination, I will advise parents to vaccinate their children against varicella*	73	81.1% [73.0–89.2]*	36	60.0% [47.6–72.4]*	109	72.7%
*I expect many questions from parents about varicella vaccination should it be recommended universally*	60	66.7% [56.9–76.4]	41	68.3% [56.6–80.1]	101	67.3%
*I have good arguments to convince parents of the importance of vaccination against varicella*	68	75.6% [66.7–84.4]*	30	50.0% [37.4–62.7]*	98	65.3%
*It will be difficult to discuss with parents about varicella vaccination should it be recommended generally*	25	27.8% [18.5–37.0]	19	31.7% [19.9–43.4]	44	29.3%

^a^Top 2 Box (4 + 5) on 5-point scale (1 = strongly disagree, 5 = strongly agree) * significant difference between GPs and pediatricians (p-value < 0.05)

Interestingly, 92% of pediatricians reported intention to comply with universal varicella vaccination, while the proportion among GPs was 77%.

A smaller proportion of respondents had the intention to advise parents to vaccinate their children against varicella if universal varicella vaccination was recommended by the Swiss NITAG as a supplementary vaccination rather than as a basic vaccination (73% vs 86%, *p* = 0.05). Most physicians (78%) agreed that a NITAG recommendation to use a measles, mumps, rubella, varicella combination vaccine (MMRV) will lead to higher vaccination rates.

Most respondents expect many questions from parents about varicella vaccination should it be recommended universally. However, less than a third stated that they would find it difficult to discuss the necessity of varicella vaccination with parents. In line with this finding, 79% of pediatricians and 68% of GPs perceived parents’ general attitude towards having their children vaccinated as positive or rather positive (additional file 2).

Remarkably, 76% of pediatricians but only 50% of GPs (*p* < 0.05) felt they will be able to convince parents of the importance of varicella vaccination (Table 2).

### Potential determinants of attitude towards universal varicella vaccination

#### Current vaccination recommendation behavior for varicella

40% of pediatricians and 27% of GPs indicated to be currently recommending varicella vaccination for all infants from the approved age (Table 3). Most physicians (64%) stated to be following the recommendation of varicella vaccination for risk groups according to the NIP and only 2 physicians responded that they are not recommending varicella vaccination to anybody (Table 3). A Mann-Whitney test indicated that the doctors who now recommend varicella vaccination for all infants showed a higher level of intended behavior to recommend UVV compared to those who recommend it only for risk groups (Mean Rank of 89.7% vs. 66.3%; *p* < 0.001).

**Table 3:** Current recommendation behavior for varicella vaccination

	Pediatricians (N = 90)		GPs (N = 60)		Total (N = 150)	
Recommendation categories	N	%	N	%	N	%
For all infants from the approved age	36	40.0%	16	26.7%	52	34.7%
Only for risk groups according to the National Immunization Plan (NIP)	53	58.9%	43	71.7%	96	64.0%
Not for anybody	1	1.1%	1	1.7%	2	1.3%

When vaccinating young children against varicella, 76 (51%) of respondents administer the MMRV combination vaccine for the first dose (52 (58%) of pediatricians, 24 (40%) of GPs) and 63 (42%) stated that they sometimes or always use separate varicella vaccination (with or without MMR in co-administration in the same visit) for the first dose (27 (30%) of pediatricians, 36 (60%) of GPs). 11 (12%) Pediatricians reported to use both MMRV combination vaccine as well as separate varicella vaccination (with or without MMR in co-administration in the same visit) for the first dose.

For the second dose, 78 (52%) respondents use MMRV (54 (60%) of pediatricians, 24 (40%) of GPs) and 52 (35%) of physicians (24 (27%) of pediatricians, 28 (47%) of GPs) indicated that they sometimes or always administer the varicella vaccine separately. 11 (12%) Pediatricians reported to use both MMRV combination vaccine as well as separate varicella vaccination (with or without MMR in co-administration in the same visit) for the second dose and 9 physicians (6%) stated to not give a second dose (1 pediatrician, 8(13%) GPs).

#### Knowledge about varicella

Based on the knowledge score, 132 (88%) physicians had a ‘moderate’ or ‘good knowledge’ about varicella in general (Table 4). The relationship between varicella and herpes zoster was known to most respondents.

**Table 4:** Knowledge of physicians about varicella zoster virus (VZV)

	Pediatricians (N = 90)		GPs (N = 60)		Total (N = 150)		
Questionnaire Item	N	% [95% CI]	N	% [95% CI]	N	%	*p*-value*
*In general, you will get varicella only once in your lifetime*							
Right (correct answer)	84	93.3% [88.2–98.5]	46	76.7% [65.9–87.4]	130	86.7%	**0.003**
Wrong	5	5.6% [0.8–10.3]	14	23.3% [12.6–34.0]	19	12.7%	
Don’t know	1	1.1%	0	0.0	1	0.7%	
*The main transmission path of varicella is a contact with fresh varicella blisters*							
Right (correct answer)	49	54.4% [44.2–64.7]	39	65.0% [52.9–77.1]	88	58.7%	0.135
Wrong	41	45.6% [35.3–55.9]	20	33.3% [21.4–45.3]	61	40.7%	
Don’t know	0	0.0	1	1.7%	1	0.7%	
*Some potential complications of varicella require the use of antibiotics*							
Right (correct answer)	80	88.9% [82.4–95.4]	41	68.3% [56.6–80.1]	121	80.7%	**0.002**
Wrong	8	8.9% [3.0–14.8]	13	21.7% [11.2–32.1]	21	14.0%	
Don’t know	2	2.2%	6	10.0%	8	5.3%	
*You can only get herpes zoster if you had a varicella infection*							
Right (correct answer)	72	80.0% [71.7–88.3]	36	60.0% [47.6–72.4]	108	72.0%	**0.008**
Wrong	16	17.8% [9.9–25.7]	20	33.3% [21.4–45.3]	36	24.0%	
Don’t know	2	2.2%	4	6.7%	6	4.0%	
*Currently, what is the hospitalization rate due to varicella in children up to the age of 16 years in Switzerland?*							
0.1 per 10′000 cases	12	13.3% [6.3–20.4]	11	18.3% [8.5–28.1]	23	15.3%	
1 per 10′000 cases	35	38.9% [28.8–48.9]	34	56.7% [44.1–69.2]	69	46.0%	
10 per 10′000 cases (correct answer)	36	40.0% [29.9–50.1]	14	23.3% [12.6–34.0]	50	33.3%	0.034
100 per 10′000 cases	7	7.8% [2.2–13.3]	1	1.7% [−1.6–4.9]	8	5.3%	
*Currently, what percentage of children in Switzerland experiences varicella before the age of 11 years?*							
50%	7	7.8% [2.2–13.3]	7	11.7% [3.5–19.8]	14	9.3%	
75%	46	51.1% [40.8–61.4]	31	51.7% [39.0–64.3]	77	51.3%	
95% (correct answer)	37	41.1% [30.6–51.3]	22	36.7% [24.5–48.9]	59	39.3%	0.585
varicella knowledge score (max 6 points) - rescaled**							**0.001**
0–2 - Limited knowledge	4	4.4%	14	23.3%	18	12.0%	
3–4 - Moderate knowledge	59	65.6%	38	63.3%	97	64.7%	
5–6 - Good knowledge	27	30.0%	8	13.3%	35	23.3%	

*Pearson’s χ^2^ or Fisher’s exact test; *p*-values in bold indicate results that are considered statistically significant after correction for multiple testing by the Benjamini–Hochberg method at a false discovery rate of 0.05

**varicella knowledge score: sum of the first 6 items above where each correct answer was awarded with 1 point, a wrong or missing answer with 0 points

Of the participating physicians, 51% believed that 75% of children in Switzerland have had varicella before the age of 11 years and thus underestimated the actual proportion, which is 95%.

Also, the hospitalization rate in children under 16 years due to varicella was underestimated with 61% of physicians assuming 0.1 or 1 per 10′000 cases, while the actual rate is 10 per 10′000 cases.

A Mann-Whitney test indicated that based on the knowledge score pediatricians showed a higher level of knowledge compared to GPs (mean of 3.98 vs. 3.00 on a scale from 0 to 6, *p* < 0.001).

#### Beliefs about varicella and varicella vaccination

When asked to rank the importance of vaccination for children for several diseases including tetanus, poliomyelitis, pertussis, measles, mumps, rubella, invasive pneumococcal disease, invasive meningococcal disease, *Haemophilus influenzae* type b infections (Hib), diphtheria, human papillomavirus (HPV), hepatitis B, tick-borne encephalitis (TBE), influenza, rotavirus and varicella, physicians considered varicella vaccination to be among the three least important vaccinations (after rotavirus and influenza). Nevertheless, about half of the participating physicians perceived it to be important or very important to vaccinate children against varicella. Pediatricians attributed a higher importance to varicella vaccination compared to GPs (Table 5).

**Table 5:** Perceived importance of specific vaccinations for children and adolescents as important or very important

	Pediatricians (N = 90)			GPs (N = 60)			Total (N = 150)		
Questionnaire Item (sorted on Total)	N	%	Mean score	N	%	Mean score	N	%	Mean score
	rating 5 or 4 (very) important			rating 5 or 4 (very) important			rating 5 or 4 (very) important		
Measles	90	100.0%	4.98	59	98.3%	4.88	149	99.3%	4.94
Tetanus	88	97.8%	4.96	60	100.0%	4.98	148	98.7%	4.97
Poliomyelitis	88	97.8%	4.81	58	96.7%	4.8	146	97.3%	4.81
*Haemophilus influenzae* type b infections	90	100.0%	4.96	56	93.3%	4.63	146	97.3%	4.83
Whooping cough (Pertussis)	89	98.9%	4.92	56	93.3%	4.77	145	96.7%	4.86
Rubella	86	95.6%	4.87	58	96.7%	4.82	144	96.0%	4.85
Mumps	85	94.4%	4.7	56	93.3%	4.73	141	94.0%	4.71
Diphtheria	84	93.3%	4.71	57	95.0%	4.7	141	94.0%	4.71
Invasive Pneumococcal infections	89	98.9%	4.88	52	86.7%	4.33	141	94.0%	4.66
Human papillomavirus	85	94.4%	4.59	52	86.7%	4.25	137	91.3%	4.45
Invasive Meningococcal infections	85	94.4%	4.73	50	83.3%	4.2	135	90.0%	4.52
Hepatitis B	79	87.8%	4.58	47	78.3%	4.23	126	84.0%	4.44
Tick-borne encephalitis	70	77.8%	4.19	45	75.0%	4.05	115	76.7%	4.13
Chickenpox (Varicella)	51	56.7%	3.61	28	46.7%	3.38	79	52.7%	3.52
Influenza	30	33.3%	2.93	19	31.7%	2.95	49	32.7%	2.94
Rotavirus	19	21.1%	2.58	11	18.3%	2.58	30	20.0%	2.58

**Fig. 1:**
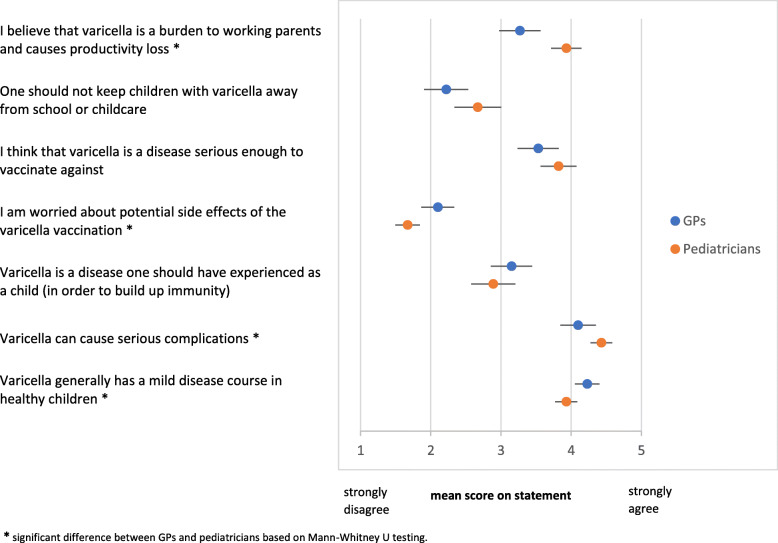
Mean score on statements regarding the beliefs about the disease varicella and varicella vaccination

Although most physicians felt that varicella generally has a mild disease course in healthy children, most respondents acknowledged that it can cause serious complications. Accordingly, there was agreement that varicella is a disease serious enough to vaccinate against (pediatricians: mean score of 3.53 and 67% either agreeing or agreeing strongly, GPs: mean score of 3.82 and 55% agreeing or agreeing strongly). Only a minority believed that children should experience varicella to develop specific natural immunity (Fig. 1).

#### Logistic regression analysis

Four determinants were statistically significantly associated with a high intention to advise parents to vaccinate their children against varicella if universal varicella vaccination was recommended by the Swiss NITAG in the multivariable logistic regression analysis (Table 6).

**Table 6:** Determinants for a high intention to advise parents to vaccinate their children against varicella if universal varicella vaccination was recommended by the Swiss NITAG from univariable and multivariable logistic regression analysis

		Physicians (Pediatricians and GPs combined)							
								95% C.I.for OR^h^	
Significant determinants	*N*^*b*^	B^c^	S.E.^d^	Wald^e^	df^f^	Sig.ª^g^	Exp(B) = OR	Lower	Upper
**Specialty**									
Pediatrician	90	1.129	0.455	6.151	1	0.013	3.091	1.267	7.542
GP	60	*Reference*							
**Beliefs about varicella and varicella vaccination**									
*Varicella is a disease one should have experienced as a child (in order to develop immunity)*				17.038	2	0.000			
No agreement	54	*Reference*							
Neutral	31	−2.707	0.656	17.038	1	0.000	0.067	0.018	0.241
Agreement	65	−1.356	0.566	5.737	1	0.017	0.258	0.085	0.782
*I think that varicella is a disease serious enough to vaccinate against*				21.432	2	0.000			
No agreement	31	*Reference*							
Neutral	26	−1.097	0.701	2.447	1	0.118	0.334	0.084	1.320
Agreement	93	1.731	0.538	10.366	1	0.001	5.644	1.968	16.185
**Years in practice**		−0.097	0.032	9.295	1	0.002	0.908	0.853	0.966
Constant		1.714	0.792	4.691	1	0.030	5.554		

a. Variable(s) entered: q13sc_3. Varicella is a disease one should have experienced as a child (in order to build up immunity), q13sc_5. I think that varicella is a disease serious enough to vaccinate against, s2. Specialty, s4. Years in practice

^b^N – base for each variable

^c^B – unstandardized regression weight

^d^S.E.– standard deviation

^e^Wald –test statistic for the individual predictor variable to determine the p value

^f^df – degrees of freedom

^g^Sig. – p-value

^h^C.I. – lower confidence limits (Lower) and the upper confidence limits (Upper)

First, physicians who are neutral or who agreed with the statement “*Varicella is a disease one should have experienced as a child*” had a lower probability of intending to recommend UVV in case of a NITAG recommendation in comparison to physicians who disagreed (OR = 0.067 and 0.258, respectively). Second, respondents supporting the statement “*I think that varicella is a disease serious enough to vaccinate against”* had a higher intention to follow a potential UVV recommendation than respondents who disagreed (OR = 5.644).

Furthermore, the analysis revealed that the medical specialty as well as fewer years in practice were associated with a positive attitude towards recommending UVV in a statistically significant way: Being a pediatrician increased the probability of having a positive attitude towards a UVV recommendation (OR = 3.091). For physicians with more years of experience – usually older physicians – the probability of having a positive attitude toward a UVV recommendation was lower (OR = 0.908).

While the knowledge score was not a significant determinant in the multivariable logistic regression analysis, a Mann-Whitney test indicated that respondents with a positive attitude towards UVV recommendation showed a higher level of knowledge compared to respondents with a negative attitude (*p* < 0.032).

There was no association between the attitude towards UVV and correct assessment of the hospitalization rate (*p*-value = 0.458).

## Discussion

Health care professionals are one of the strongest influencers in vaccination decisions [16, 17] and therefore critically influence whether a recommended vaccination reaches high coverage in the population.

In the event the Swiss NITAG would recommend UVV, most participating physicians state that they would advise parents to have their children vaccinated. This expression of compliance was 92% among pediatricians and 77% among GPs. As pediatricians in Switzerland are the first-line providers of primary care in the early years of life [18], the finding that more than 90% of pediatricians in our study showed a high intention to recommend UVV in case of a NITAG recommendation indicates that it should be feasible to achieve high vaccination coverage rates.

The high acceptance of an UVV recommendation in our study is supported by the finding that already more than a third of respondents – predominantly pediatricians – currently recommend varicella vaccination for all infants. It can be speculated that this recommendation behavior is influenced by the experience of nearby countries with universal varicella vaccination programs in place, such as Germany, Italy and Spain [7, 9, 10].

It is also reassuring that most participating physicians indicated that they have strong arguments to educate parents on the importance of vaccination against varicella.

In line with this finding, about three quarters of the participating physicians perceived parents’ attitude towards having their children vaccinated as rather positive.

Nevertheless, further information and education of physicians on varicella should be considered to accompany the potential introduction of UVV, especially among the GP population who, according to the results of this study, seem to have a lower awareness of the burden of varicella.

Other recent studies investigated the attitudes to vaccinate against varicella among health care professionals and/or the public at a time when universal varicella vaccination was not recommended. A study previously conducted in the Netherlands showed that health care professionals and parents had a negative attitude or low intention to vaccinate universally against varicella, as a result of the perceived low severity of the disease. Accordingly, there was also very low demand for varicella vaccines in the private market with only ~ 165 varicella vaccines for children below 5 years delivered by Dutch community pharmacies in 2014 [14]. In contrast, a small qualitative survey study among 20 caregivers and providers in New Zealand revealed positive support towards universal varicella vaccination and a high intention to vaccinate if available as a routine vaccine [19]. The results of our survey study are thus more comparable to those observed in New Zealand than in the Netherlands.

Other survey studies have focused on parents, rather than on providers. One year after the introduction of a UVV program in Naples, Italy, a survey among 675 parents showed that less than 27% of parents knew that varicella vaccination was available and that the perceived utility of varicella vaccination was low. Importantly, the positive attitude towards the utility was however higher in those parents who had received information from a health care provider [20]. A survey among visitors of a German internet vaccine forum for lay persons conducted a few months before UVV was introduced in Germany revealed modest acceptance of varicella immunization [16]. Despite this early indication of low initial acceptance among parents, it is noteworthy that varicella vaccination coverage with 1 dose at the age of 24 months reached > 80% within 4 years after introduction of universal varicella vaccination in Germany [21].

This study has some limitations. The study sample was not fully representative of the Swiss population of pediatricians and the GPs who treat pediatric patients. Physicians from the Italian-speaking part of Switzerland, who make up 4.35% of all GPs practicing and 4.25% of all pediatricians practicing in Switzerland [22] were not included. Also, there is potential for selection bias as physicians who were willing to participate in the study might have had more favorable attitudes towards vaccination than the average Swiss pediatrician or GP. Furthermore, the outcome of this study is based on physicians’ perceptions and feedback (i.e. stated preferences) and not on a systematic analysis of patient record forms or chart review. Sampling errors could have affected the precision and interpretation of the results. The study is only a cross-sectional analysis at a specific point in time and does not allow an interpretation of developments or trends over time. The regression model analysis did not take into account all relevant confounders leading to unmeasured confounding bias. Finally, the questionnaire used for the survey study is not a standardized, validated questionnaire. Therefore, outcomes of this study are not comparable with those from other countries or studied samples.

Still, this is the first survey study among pediatricians and GPs in Switzerland specifically addressing varicella vaccination. Many aspects need to be taken into consideration in a national decision-making process about UVV, as has been outlined by a report of the European Centre for Disease Prevention and Control [23]. The findings of this survey study may assist the Swiss NITAG in its evaluations of the feasibility of UVV introduction in the national immunization schedule.

## Conclusions

The results of this survey show that most participating pediatricians and GPs indicated a favorable attitude towards childhood vaccination against varicella in the setting of a Swiss NITAG recommendation for UVV. This data also shows the importance of NITAG recommendations in influencing vaccine education and supporting achievement of high coverage of varicella vaccination.

## Supplementary Information

**Additional file 1.** Study questionnaire.

**Additional file 2.** Perceived general attitude of parents towards having their children vaccinated according to NITAG recommendations.

## Data Availability

The datasets used and/or analysed during the current study are available from the corresponding author on reasonable request.

## Abbreviations

UVV: universal varicella vaccination
NITAG: National Immunization Technical Advisory Group
GPs: general practitioners
VZV: varicella zoster virus
NIP: National Immunization Plan
MMRV: measles, mumps, rubella, varicella combination vaccine
MMR: measles, mumps, rubella combination vaccine
