# Supplementary material for: Acceptance of universal varicella vaccination among Swiss pediatricians and general practitioners who treat pediatric patients
Source: BMC Infect Dis. 2021 Jan 6;21:12. doi: 10.1186/s12879-020-05586-3 (PMC7789518; doi:10.1186/s12879-020-05586-3)
Supplement: Supplementary file 1 — Additional file 1. Study questionnaire. [file 12879_2020_5586_MOESM1_ESM.docx]

Ipsos Healthcare

**Varicella vaccination in Switzerland –**

**Quantitative Screener and Questionnaire**

**Sample quotas:**

|  |  |  | |  |
| --- | --- | --- | --- | --- |
|  | ***Respondent type*** | **D-CH**  **(ca. 3/4)** | | **W-CH**  **(ca. 1/4)** |
|  | **Pediatricians (n=80-100)** | 60-75 | | 20-25 |
|  | **GPs (n=50-70)** | 35-50 | | 15-20 |
|  | **TOTAL: N=130 - 150** |  |  | |

**Methodology: Online questionnaire**

**Interview length: 20 minutes**

INTRODUCTION **[SHOW EVERYTHING ON ONE SCREEN]**

Thank you for agreeing to participate in this survey.

This survey is being conducted by Ipsos Healthcare, an independent market research agency, on behalf of a pharmaceutical company.

We are conducting this market research about **varicella vaccination in Switzerland** and would like to ask you some questions on this topic. The survey will take about **20 minutes** of your time.

As a member of EphMRA, Ipsos Healthcare is bound by **EphMRA Code of Conduct** and all applicable laws protecting your personal data and responses. The study is conducted in compliance with ESOMAR/ EphMRA guidelines.

You have the right to withdraw from the survey at any time. Any information you give will be treated in the strictest confidence and results will only be reported back on an aggregated basis, so that we get an overall impression of the perceptions in Switzerland.

**[AE DISCLAIMER FOR HCPs:** In accordance with the European Pharmaceutical Market Research Association (EphMRA) Code of Conduct and European Medicines Agency good pharmacovigilance practice guidelines, it is our responsibility to report to the sponsoring client any adverse events or product quality complaints, on the client’s products that become known to us when conducting healthcare market research. Although what is otherwise recorded during this survey will be treated in confidence, should an adverse event (as defined in the Code of Conduct) or product quality complaint in a specific patient, or group of patients, be mentioned, we are required to collect this information and report it to the client, even if the event has already been reported to the national health authorities.

I acknowledge that I have read, understood and accepted the above points and would like to continue based on the market research survey.

☐ Yes **[CONTINUE]**

☐ No **[CLOSE]**

**[SHOW ON SEPERATE SCREEN]**

**The adverse event will be reported in such a way to maintain your confidentiality**. In some cases, the sponsoring company’s pharmacovigilance department may need additional information from you to properly classify the adverse event. In this instance the Market Research agency may be in contact, via email, for further details.

Would you agree that we may contact you again in such a case, and only in relation to the mentioned side effect? Any other information you provide in this survey will remain strictly confidential.

☐ Yes **[CONTINUE]**

☐ No **[CONTINUE]**

**SECTION LABEL: SCREENER**

S1

Single Answer

BASE: All respondents

Please specify your region:

| 1 | D-CH |
| --- | --- |
| 2 | W-CH |

S1a

Single Answer

BASE: All respondents

Please specify the canton in which you mainly practice:

| 1 | Aargau |
| --- | --- |
| 2 | Appenzell Innerrhoden |
| 3 | Appenzell Ausserrhoden |
| 4 | Bern |
| 5 | Basel-Land |
| 6 | Basel-Stadt |
| 7 | Freiburg |
| 8 | Genf |
| 9 | Glarus |
| 10 | Graubünden |
| 11 | Jura |
| 12 | Luzern |
| 13 | Neuenburg |
| 14 | Nidwalden |
| 15 | Obwalden |
| 16 | St. Gallen |
| 17 | Schaffhausen |
| 18 | Solothurn |
| 19 | Schwyz |
| 20 | Thurgau |
| 21 | Tessin |
| 22 | Uri |
| 23 | Waadt |
| 24 | Wallis |
| 25 | Zug |
| 26 | Zürich |

S2

Single Answer

BASE: All respondents

First, we'd like to ask you some general questions to check if you meet our target criteria.

What is your primary specialty?

| 1 | GP |  |
| --- | --- | --- |
| 2 | Pediatrician |  |
| 3 | Other, please specify ____ | => Screenout |

S3

Numeric answer

BASE: All respondents

In which setting do you mainly practice?

| 1 | Private practice |  |
| --- | --- | --- |
| 2 | University hospital / Cantonal hospital / Regional hospital | => Screenout |

S4

Numeric answer

BASE: All respondents

How long have you been practicing in your specialty?

___________ years => Screenout if <2 or >35

S5

Single Answer

BASE: All respondents

Please specify your gender:

| 1 | Male |
| --- | --- |
| 2 | Female |

S6

Single Answer

BASE: All respondents

Are you currently vaccinating children?

| 1 | Yes |
| --- | --- |
| 2 | No => Screenout |

S7

Single Answer

BASE: All respondents

Do you have children of your own?

| 1 | Yes | => continue to S06 |
| --- | --- | --- |
| 2 | No |  |

S8

Single Answer

BASE: Those who said they have children at S07 [S07=1]

Are your children vaccinated according to the National Immunization Plan (NIP)?

| 1 | Yes |
| --- | --- |
| 2 | No |

***Quota – please see quota table above***

S99 SCREEN-OUT TEXT

Thank you for your interest in participating in this market research study.

Unfortunately, your profile does not meet our criteria this time. Thank you for taking the time to answer our questions.

**SECTION LABEL: MAIN QUESTIONNAIRE**

Based on your answers, you fulfill the conditions for participating in this market research study, which deals with your views on **varicella vaccination in Switzerland**.

**General part** [Do not show this header to respondents]

Q1

Single answer

BASE: All respondents

To get started, we would like to learn more about your general view of vaccination in children.

For each of the statements below, please indicate to what extent you agree with them. Please use a scale of 1 to 5 on which 1 means 'not at all' and 5 means 'fully agree'.

//Scripter: Randomize//

|  |  | Strongly disagree  (1) | Somewhat disagree  (2) | Neutral  (3) | Somewhat agree  (4) | Fully agree  (5) |
| --- | --- | --- | --- | --- | --- | --- |
| 1 | The spread of certain diseases can be prevented by vaccinating people against it | 🔿 | 🔿 | 🔿 | 🔿 | 🔿 |
| 2 | When I think about vaccinating people, I always carefully weigh the benefits and risks before I vaccinate | 🔿 | 🔿 | 🔿 | 🔿 | 🔿 |
| 3 | As far as the availability of vaccines is concerned, I trust that the authorities will always decide in the best interest of the general public | 🔿 | 🔿 | 🔿 | 🔿 | 🔿 |
| 4 | I have complete confidence in the safety of vaccinations | 🔿 | 🔿 | 🔿 | 🔿 | 🔿 |
| 5 | Experiencing a childhood disease (e.g., varicella) is beneficial to a child’s development | 🔿 | 🔿 | 🔿 | 🔿 | 🔿 |

Q3

Multiple answers

BASE: All respondents

Which of the following channels do you personally think are best suited to inform people about childhood and adulthood vaccinations?

//Scripter: Randomize//

| 1 ❑ | In-person conversation with a doctor |
| --- | --- |
| 2 ❑ | Information provided by the health insurance |
| 3 ❑ | Information from the health department / authorities |
| 4 ❑ | In-person conversation with a health professional e.g. a medical assistant or a midwife |
| 5 ❑ | Information in a pharmacy |
| 6 ❑ | Information brochures or flyers |
| 7 ❑ | Information on Posters (e.g. on the street, in public transit or bus stops) |
| 8 ❑ | Information events in schools or kindergartens |
| 9 ❑ | Conversations with family members, friends or acquaintances |
| 10 ❑ | Television, e.g. TV advertising |
| 11 ❑ | Internet |
| 12 ❑ | Social Media, e.g. social networks, online forums, blogs, YouTube |
| 13 ❑ | Newspapers or magazines |
| 14 ❑ | Radio |
| 15 ❑ | Information from pharma companies |
| 99 ❑ | Other, please specify: _______________ |

Q4

Open End

BASE: Those who chose 11 at Q03

You have identified the Internet as a suitable source of information for childhood and adulthood vaccines. Can you name specific websites that you think of as good examples?

_________________________

Q5

Single answer

BASE: All respondents

How do you feel about the general attitude of the parents in Switzerland towards having their children vaccinated? In your opinion, they are rather…

| 2 ❑ | ... positive |
| --- | --- |
| 2 ❑ | ... somewhat postive |
| 3 ❑ | ... part positive, part negative |
| 4 ❑ | ... rather negative |
| 5 ❑ | ... negative |

Q6

Single answer

BASE: All respondents

Do you generally check the completeness of recommended vaccinations in your young patients during the consultation hours?

| 1 ❑ | Yes, always |
| --- | --- |
| 2 ❑ | Yes, but only for some children |
| 3 ❑ | No, that’s the job of the parents |

Q7

Multiple answers

BASE: All respondents

Which of the following measures do you usually take when it comes to reminding people of their pending vaccination dates?

| 1 ❑ | I remind them in person during their consultation |
| --- | --- |
| 2 ❑ | I use vaccination information (posters, leaflets, brochures) available in my practice |
| 3 ❑ | I remind them per mail, e-mail, a telephone call or text message |
| 4 ❑ | I give a personal vaccination calendar to the parents |
| 5 ❑ | I don’t take any measures to remind patients of vaccinations |
| 6 ❑ | Other, please specify ___________________ |

**Current vaccination behaviour** [Do not show this header to respondents]

In the following section we would like to better understand the current vaccination behavior of physicians in Switzerland.

Q8

Single answer

BASE: All respondents

Please indicate to what extent you agree with the following statement.

Please use a scale from 1 to 5, where 1 means strongly disagree' and 5 means 'fully agree'.

I currently advise parents to vaccinate their infants against **pneumococcal disease** according to the NIP.

| Strongly disagree  (1) | Somewhat disagree  (2) | Neutral  (3) | Somewhat agree  (4) | Fully agree  (5) |
| --- | --- | --- | --- | --- |
| 🔿 | 🔿 | 🔿 | 🔿 | 🔿 |

Q9

Multiple answer EXCEPT if option 3 is chosen

BASE: All respondents

Now please think about the varicella vaccination specifically. For whom do you currently recommend this vaccination?

| 1 ❑ | For all infants from the approved age |
| --- | --- |
| 2 ❑ | Only for risk groups according to the National Immunization Plan (NIP) |
| 98 🔿 | I wouldn’t recommend it for anybody |

Q9a

Open End

BASE: All respondents

Please let us know why you say so:

**_________________________________________________________**

Q10

Multiple answer

BASE: All respondents

If you vaccinate infants against varicella, which type of vaccine do you use for the **first dose**?

| 1 ❑ | V (with or without MMR in parallel) |
| --- | --- |
| 2 ❑ | MMRV |

Q11

Multiple answer

BASE: All respondents

If you vaccinate infants against varicella, which type of vaccine do you use for the **second dose**?

| 1 ❑ | V (with or without MMR in parallel) |
| --- | --- |
| 2 ❑ | MMRV |
| 3 ❑ | I don’t give a second dose |

**Knowledge about the varicella zoster virus** [Do not show this header to respondents]

Q12

Single answer per row

BASE: All respondents

Please indicate for each of the statements below whether you believe it to be true or false.

*//SCRIPTER: RANDOMIZE//*

|  |  | **False** | **True** | **Don’t Know** |
| --- | --- | --- | --- | --- |
| 1 | In general, you will get varicella only once in your lifetime | 🔿 | 🔿 | 🔿 |
| 2 | The main transmission path of varicella is a contact with fresh varicella blisters | 🔿 | 🔿 | 🔿 |
| 3 | Some potential complications of varicella disease require the use of antibiotics | 🔿 | 🔿 | 🔿 |
| 4 | You can only get herpes zoster if you had a varicella infection | 🔿 | 🔿 | 🔿 |
| 5 | Repeated exposure to VZV boosts the immune system resulting in a lower risk for developing Herpes Zoster | 🔿 | 🔿 | 🔿 |

Q12a

Single answer

BASE: All respondents

For each of the questions below, please give your best guess by selecting one of the four answer options:

|  |  | **50%** | **75%** | **95%** | **100%** |
| --- | --- | --- | --- | --- | --- |
| 1 | Currently, what percentage of children in Switzerland experiences varicella before the age of 11years? | 🔿 | 🔿 | 🔿 | 🔿 |

Q12b

Single answer

BASE: All respondents

|  |  | **0.1 per 10'000 cases** | **1 per 10'000 cases** | **10 per 10'000 cases** | **100 per 10'000 cases** |
| --- | --- | --- | --- | --- | --- |
| 1 | Currently, what is the hospitalization rate due to varicella in children up to the age of 16 years in Switzerland? | 🔿 | 🔿 | 🔿 | 🔿 |

**Perceptions of Varicella and the Varicella vaccination** [Do not show this header to respondents]

Q13

Single answer

BASE: All respondents

For each of the statements below, please indicate to what extent you agree. Please use a scale of 1 to 5 on which 1 means strongly disagree' and 5 means 'fully agree'.

*//SCRIPTER: RANDOMIZE//*

|  |  | Strongly disagree  (1) | Somewhat disagree  (2) | Neutral  (3) | Somewhat agree  (4) | Fully agree  (5) |
| --- | --- | --- | --- | --- | --- | --- |
| 1 | Varicella generally has a mild disease course in healthy children | 🔿 | 🔿 | 🔿 | 🔿 | 🔿 |
| 2 | Varicella can cause serious complications | 🔿 | 🔿 | 🔿 | 🔿 | 🔿 |
| 3 | Varicella is a disease one should have experienced as a child (in order to build up immunity) | 🔿 | 🔿 | 🔿 | 🔿 | 🔿 |
| 4 | I am worried about potential side effects of the varicella vaccination | 🔿 | 🔿 | 🔿 | 🔿 | 🔿 |
| 5 | I think that varicella is a disease serious enough to vaccinate against | 🔿 | 🔿 | 🔿 | 🔿 | 🔿 |
| 6 | One should not keep children with varicella away from school or child care | 🔿 | 🔿 | 🔿 | 🔿 | 🔿 |
| 7 | I believe that varicella disease is a burden to working parents and causes productivity loss | 🔿 | 🔿 | 🔿 | 🔿 | 🔿 |

Q14

Single answer

BASE: All respondents

For each of the statements below, please indicate to what extent you agree. Please use a scale of 1 to 5 on which 1 means 'strongly disagree' and 5 means 'fully agree'.

*//SCRIPTER: RANDOMIZE//*

|  |  | Strongly disagree  (1) | Somewhat disagree  (2) | Neutral  (3) | Somewhat agree  (4) | Fully agree  (5) |
| --- | --- | --- | --- | --- | --- | --- |
| 1 | If the BAG/EKIF recommends varicella vaccination for all infants starting at the age of 9 (-12) months as a basic vaccination, I will advise parents to vaccinate their children against varicella. | 🔿 | 🔿 | 🔿 | 🔿 | 🔿 |
| 2 | If the BAG/EKIF recommends varicella vaccination for all infants starting at the age of 9(-12) months as a supplementary vaccination, I will advise parents to vaccinate their children against varicella | 🔿 | 🔿 | 🔿 | 🔿 | 🔿 |
| 3 | A strong recommendation from BAG/EKIF to use the quadrivalent measles, mumps, rubella, varicella (MMRV) will lead to higher vaccination rates | 🔿 | 🔿 | 🔿 | 🔿 | 🔿 |
| 4 | I expect many questions from parents about varicella vaccination should it be recommended generally | 🔿 | 🔿 | 🔿 | 🔿 | 🔿 |
| 5 | It will be difficult to discuss with parents about varicella vaccination should it be recommended generally | 🔿 | 🔿 | 🔿 | 🔿 | 🔿 |
| 6 | I have good arguments to convince parents of the importance of vaccination against varicella | 🔿 | 🔿 | 🔿 | 🔿 | 🔿 |

Q2

Single answer

BASE: All respondents

Finally, we would like to ask you a question about other vaccinations.

For each of the diseases listed below, please indicate the importance of vaccination for children (from the approved age onwards) – according to your opinion.

Please use a scale of 1 to 5 on which 1 means ‘not very important’ and 5 means ‘very important’.

|  |  | Not very important  (1) | (2) | (3) | (4) | Very important  (5) |
| --- | --- | --- | --- | --- | --- | --- |
| 1 | Tetanus | 🔿 | 🔿 | 🔿 | 🔿 | 🔿 |
| 2 | Polio (Poliomyelitis, IPV) | 🔿 | 🔿 | 🔿 | 🔿 | 🔿 |
| 3 | Measles | 🔿 | 🔿 | 🔿 | 🔿 | 🔿 |
| 4 | Hepatitis B (HBV) | 🔿 | 🔿 | 🔿 | 🔿 | 🔿 |
| 5 | Rubella | 🔿 | 🔿 | 🔿 | 🔿 | 🔿 |
| 6 | Mumps | 🔿 | 🔿 | 🔿 | 🔿 | 🔿 |
| 7 | Whooping cough (Pertussis) | 🔿 | 🔿 | 🔿 | 🔿 | 🔿 |
| 8 | Chickenpox (Varicella) | 🔿 | 🔿 | 🔿 | 🔿 | 🔿 |
| 9 | Diphtheria | 🔿 | 🔿 | 🔿 | 🔿 | 🔿 |
| 10 | Haemophilus-influenzae-Type-b-infections (Hib) | 🔿 | 🔿 | 🔿 | 🔿 | 🔿 |
| 11 | Invasive Pneumococcal infections | 🔿 | 🔿 | 🔿 | 🔿 | 🔿 |
| 12 | Invasive Meningococcal infections | 🔿 | 🔿 | 🔿 | 🔿 | 🔿 |
| 13 | Rotavirus | 🔿 | 🔿 | 🔿 | 🔿 | 🔿 |
| 14 | Tick-borne encephalitis (FSME) | 🔿 | 🔿 | 🔿 | 🔿 | 🔿 |
| 15 | Influenza | 🔿 | 🔿 | 🔿 | 🔿 | 🔿 |
| 16 | Human papillomavirus (HPV) | 🔿 | 🔿 | 🔿 | 🔿 | 🔿 |
| 98 | Other, please specify: [Insert open text box]_________ | 🔿 | 🔿 | 🔿 | 🔿 | 🔿 |

Q15

Multiple answers EXCEPT IF 98 (Not interested) is chosen

BASE: All respondents

We are now almost at the end of the survey.

For which vaccination topics would you like to receive additional information in the future?

| 1 ❑ | Side effects and health risks of vaccinations |
| --- | --- |
| 2 ❑ | Reimbursement of vaccinations by the health insurance companies |
| 3 ❑ | Updated vaccination recommendations of the BAG |
| 4 ❑ | New developments in the field of vaccines |
| 5 ❑ | Informational material (e.g., brochures) about vaccinations for my patients |
| 98 🔿 | No interest |
| 99 ❑ | Other, please specify: _______________ |

[NEXT SCREEN]

Finally, the correct answers for the knowledge questions earlier:

|  |  | **False** | **True** | **Don’t Know** |
| --- | --- | --- | --- | --- |
| 1 | In general, you will get varicella only once in your lifetime |  | **X** |  |
| 2 | The main transmission path of varicella is a contact with fresh varicella blisters | **X** |  |  |
| 3 | Some potential complications of varicella disease require the use of antibiotics |  | **X** |  |
| 4 | You can only get herpes zoster if you had a VZV infection (varicella) |  | **X** |  |
| 5 | Repeated exposure to VZV boosts the immune system and may result in a lower risk for developing Herpes Zoster |  | **X** |  |

What percentage of children in Switzerland have had varicella before the age of 11??

A: __**95%**__

What is the hospitalization rate due to chickenpox in children under the age of 16 in Switzerland?

A: __ **10 per 10'000 cases** ___

*Reference: Bonhoeffer J et al. Prospective surveillance of hospitalisations associated with varicella-zoster virus infections in children and adolescents Eur J Pediatr (2005) 164: 366–370*

Thank you for your participation in our survey!
