# Supplementary material for: Acceptance of universal varicella vaccination among Swiss pediatricians and general practitioners who treat pediatric patients
Source: BMC Infect Dis. 2021 Jan 6;21:12. doi: 10.1186/s12879-020-05586-3 (PMC7789518; doi:10.1186/s12879-020-05586-3)
Supplement: Supplementary file 2 — Additional file 2. Perceived general attitude of parents towards having their children vaccinated according to NITAG recommendations. [file 12879_2020_5586_MOESM2_ESM.docx]

**Additional file 2** Perceived general attitude of parents towards having their children vaccinated according to NITAG recommendations

|  | Pediatricians (N=90) | |  | GPs (N=60) | |  | Total (N=150) | |
| --- | --- | --- | --- | --- | --- | --- | --- | --- |
| Perceived attitude of parents towards vaccination | N | % |  | N | % |  | N | % |
|  |  |  |  |  |  |  |  |  |
| Positive | 14 | 15.6% |  | 3 | 5.0% |  | 17 | 11.3% |
| Rather positive | 57 | 63.3% |  | 38 | 63.3% |  | 95 | 63.3% |
| Partly positive | 18 | 20.0% |  | 19 | 31.7% |  | 37 | 24.7% |
| Rather negative | 1 | 1.1% |  | 0 | 0.0% |  | 1 | 0.7% |
| Negative | 0 | 0.0% |  | 0 | 0.0% |  | 0 | 0.0% |
